# On Vapour Baths

**Published:** 1805-06-01

**Authors:** 


### To Dr. BRADLEY.

SIR,

IF the following observations meet with your approbation, I will thank you to give them a place in the Medical Journal. They were suggested by a perusal of Dr. Reid's paper on the Vapour Baths of Russia; but are not intended as an answer to that gentleman's query, which is left for the solution of the learned and ingenious proposer, whose practical skill and acumen I have frequently observed with pleasure.

Vapour baths are in very general use among the northern nations, either as articles of luxury, or as preventives or cures of disease. The Esquimaux, the Icelanders, and the Kamschatdales, delight in the use of stoves, heated perhaps to a greater degree than those of Russia. The North American can Indian, when attacked by sickness, converts his wigwam into a vapour bath, and, when hissing hot, plunges into a river through a hole in the ice. No doubt can be entertained of the safety of the practice, nor, from its general use, of its salubrity. It would appear almost as if suggested by instinct, in such high latitudes, to obviate that degree of constriction on the surface, occasioned by long-continued cold, by supporting the balance between the external and internal circulation, and thus preserving the vessels of the skin in a permeable state.

This practice of alternating extreme degrees of heat and cold, appears, to a delicate person, fraught with danger; and he experiences a sensation of horror from the bare idea of feeling, "by turns, the bitter change

> Of fierce extremes, extremes by change more fierce,
> From beds of raging fire to starve in ice."

The habit of bearing these changes, so as to derive pleasure from them, is laid in early infancy, and continued through life, at regular periods. Mr. Taunton experienced a violent smarting sensation on the skin, during his stay in the bath, and appears to have been a good deal incommoded by the heat, though, from the state of his pulse, previously to his entering the bath, I should not suspect him to be of an irritable habit. A similar sensation is felt on entering a salt-pan when the boilers are at work; and few people enter these places, for the first time, without drawing back as soon as the steam strikes the face; the heat is extremely pungent, and gives the sensation of flaying.

The constitution of the common Russian is naturally robust, and possessed of a low degree of sensibility; his nerves are of iron, and his functions are regulated by mechanism. This might be inferred from the horrid punishment of the knout, which none but a Russian could survive; and from organs of digestion, equal to those of an ostrich, and which can bear with as little annoyance a bumper of brandy or of train oil. To a Russian, brandy and the hot bath constitute the "Panem and Circenses;" they are strongly impressed with their salubrity, and never fail, in sickness, to have recourse to these Herculean remedies. Many physicians have celebrated the Russian baths; and have even ascribed the comparative mildness of the small pox in Russia to their use, especially Sanches, in a treatise, "De cura variolarum vaporarii ope apud Russos, omni memoria antiquioris, usu recepti." Dr. Currie, in his valuable Medical Reports, ascribes the safety and agreeableness of these sudden transitions merely to an increase of the heat of of the body above the natural standard; but it appears to me more just, to ascribe it to the increased activity of the cutaneous vessels. These are powerfully stimulated by heat; and in habits such as those under consideration, where the contractile power of the muscular fibre is great, the vascular action is not soon overcome. This action of the cutaneous vessels the Russians endeavour to increase artificially, by gently flagellating themselves with bunches of twigs, or leafy branches, which occasionally also serve the purpose of fig-leaves during this assemblage of the sexes; and when, by this mode of irritation, a considerable degree of erythema is excited upon the skin, they plunge without fear into the coldest streams.

In hot climates, where the body is relaxed by long exposure to an uniformly high degree of heat, an incautious exposure to a stream of air, but a few degrees perhaps below the temperature of the body, is not unfrequently followed by alarming consequences. The morbid effects, in this instance, neither arise from the retention of any thing acrimonious, nor from diminution of temperature, but from the sudden disturbance of the circulating mass. The atonic state of the cutaneous vessels is so great, that it can scarcely be called action; a slight degree of cold, especially if assisted by moisture, produces immediate constriction, and a sudden determination of the circulating mass to the interior parts, which have been long in a comparative state of depletion, and are now unable to bear so sudden a shock. The effects produced by this change are various: when the determination is made to the chylopoetic viscera, and especially to the liver, cholera most commonly ensues; when, to the whole extent of the bowels, diarrhœa; and when to a smaller portion, dysentery. These effects also more readily ensue, according as each part has been weakened by previous attacks. In our changeable climate we frequently observe the vibrating balance between the skin and the kidneys; a person going into a cold room feels a desire to void his urine; or a flow is made to the pituitary membrane inducing coryza; or to the lax and spongy tonsils, inducing cynanche. If these effects do not follow exposure to much higher degrees of cold in Russia, we must attribute it to the same cause which renders them strangers to what we call nervous complaints. Nor does it appear strange, upon reflection, that pleasure should be derived from the alternation of high degrees of heat and cold; because when the skin is in the proper state of excitement, it seems probable that the the shock communicated to it will be only equal to what it would have experienced from the mean degree of temperature in the ordinary state of excitement.

*Jan.* 11, 1805. MEDICUS.

